# Demonstration of a two-bit controlled-NOT quantum-like gate using classical acoustic qubit-analogues

**DOI:** 10.1038/s41598-022-18314-5

**Published:** 2022-08-18

**Authors:** Keith Runge, M. Arif Hasan, Joshua A. Levine, Pierre A. Deymier

**Affiliations:** 1 Department of Materials Science and Engineering, The University of Arizona, Tucson, AZ 85721 USA; 2 Department of Mechanical Engineering, Wayne State University, Detroit, MI 48202 USA; 3 Department of Computer Science, The University of Arizona, Tucson, AZ 85721 USA

**Keywords:** Acoustics, Information theory and computation

## Abstract

The Controlled-NOT (CNOT) gate is the key to unlock the power of quantum computing as it is a fundamental component of a universal set of gates. We demonstrate the operation of a two-bit C-NOT quantum-like gate using classical qubit acoustic analogues, called herein logical phi-bits. The logical phi-bits are supported by an externally driven nonlinear acoustic metamaterial composed of a parallel array of three elastically coupled waveguides. A logical phi-bit has a two-state degree of freedom associated with the two independent relative phases of the acoustic wave in the three waveguides. A simple physical manipulation involving the detuning of the frequency of one of the external drivers is shown to operate on the complex vectors in the Hilbert space of pairs of logical phi-bits. This operation achieves a systematic and predictable C-NOT gate with unambiguously measurable input and output. The possibility of scaling the approach to more phi-bits is promising.

Early proposals for a quantum mechanical computer^1,2^ emphasized the computation process, and in particular the logic gates that carry operations. These gates are reversible unitary operations acting on an input, e.g., state or superposition of states of quantum bits (qubits), producing a predictable and possibly measurable output state. The realization of a quantum controlled-NOT or C-NOT gate, a two-bit gate, has been highly desirable since it can form a universal set of gates in conjunction with a small number of single bit gates. This fundamental quantum logic gate has been demonstrated for quantum bits stored in the degrees of freedom of trapped atoms^3–5^, trapped ions^6,7^, superconducting qubits^8,9^, photons^10,11^, solid-state spins^12–14^ and proposed in topological quantum bits^15^.

The advantage of qubits over conventional bits results from the properties of their quantum wave functions, i.e., probability amplitudes, which can support coherent superpositions of multipartite states (e.g., entangled states). However, quantum computing with multiple bits suffers from the fragility of quantum superpositions of states against perturbations or undesired interactions. Increasing the performance of quantum gates, therefore, requires cumbersome strategies for suppressing environmental effects^12,16^ and performing error corrections^17^. Very recently, researchers also proposed the realization of multi-qubit controlled nonadiabatic holonomic gates with connecting systems^18^. In contrast, acoustic waves have been shown recently to also support coherent superpositions (e.g., classically entangled states) that are robust since classical wave functions are amplitudes^19,20^. This notion of “classical entanglement” possesses the non-separability and related complexity that are essential to reach the promise of parallelism in quantum computing, but not the nonlocal aspect of quantum entanglement^21,22^. Since quantum computing harnesses the non-separability of entangled states, non-separable classical waves may then serve as a resource to complement quantum systems in harnessing the power of complexity in information processing.

To that effect, the physical acoustic phase-bit or phi-bit^19^ in acoustic metamaterials was introduced as a classical analogue of a qubit. Specifically, a phi-bit associates with a two-state degree of freedom of an acoustic wave, which can be in a coherent superposition of states with complex amplitude coefficients. We recently expanded the notion of phi-bits from the physical to the logical realm^23^. Logical phi-bits exploit the strong coupling and nonlinearity of acoustic waves to realize non-separable superpositions of states spanning exponentially complex spaces (i.e., Hilbert spaces), a prerequisite to develop algorithms that exploit the computational parallelism arising from non-separability, and hence can be employed for programming.

When driven externally with two different frequencies, a metamaterial composed of an array of three elastically coupled acoustic waveguides, produces a nonlinear displacement field which can be partitioned in the frequency domain^23^. Each waveguide consists of a finite length aluminum rod with circular cross section. The rods are arranged in a linear array with a lateral gap filled with epoxy. Ultrasonic transducers drive and detect the acoustic field at the ends of the rods. Function generators and amplifiers are used to excite two driving transducers attached to the ends of guide 1 and 2 with sinusoidal signals with frequency $f_{1}$ and $f_{2}$. Three detecting transducers located at the opposite ends of the rods collect data on the displacement field. The nonlinearity of this driven system leads to many ways of mixing the drivers’ frequencies. The measured displacement field at the detection end of the waveguides is the Fourier sum of a large number of linear and nonlinear modes, each with its own characteristic frequency. The nonlinear modes, that is the phi-bits, are correlated through the nonlinear interactions of the waveguide-transducer-amplifier-generator assembly. Further details on this physical system can be found in Ref.^23^.

Subsequently, a logical phi-bit is defined as a two-level nonlinear mode of vibration whose state is characterized by a nonlinearly mixed frequency and spatial mode associated with two independent relative phases of the displacement between the waveguides. Since the phi-bits co-locate within the same physical space they are subjected to distance independent interactions. Recently, we have shown experimentally and theoretically that tensor product structures of systems comprising large numbers of logical phi-bits (*P* = 16) can support non-separable states in scalable exponentially complex Hilbert spaces^23^. Non-separability was identified by the nonzero von Neumann entropies of various partitioning of the 16 phi-bit system. Details of this physical system and the characteristics (nonlinear frequency, amplitude) of the 16 logical phi-bits are given in Ref.^23^.

In this communication we demonstrate experimentally that one can navigate in a controllable manner the Hilbert space of pairs of logical phi-bits to achieve a systematic and predictable C-NOT gate with unambiguously measurable input and output. This work establishes the foundations for acoustic quantum-like gates.

## Results

**Figure 1:**
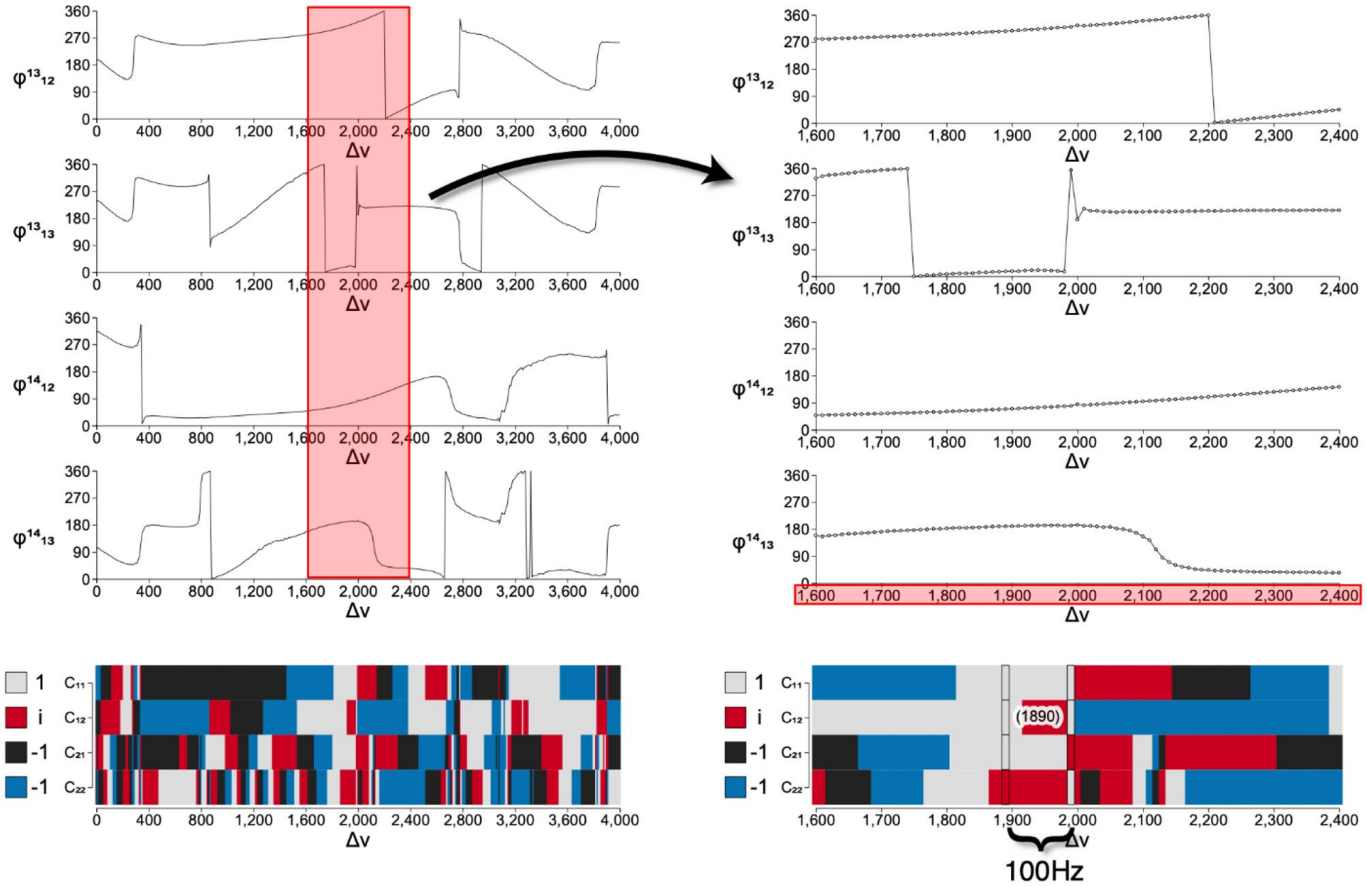
Analyzing two level states of logical phi-bits. Example of measured variations of the relative phases $\varphi_{12}^{i}$ and $\varphi_{13}^{i}$ for phi-bits *i* = 13, 14 are shown on the top left. The phases are in degrees, and the tuning parameter $\Delta \nu$ is in Hz. On the top right figure we focus on a region between 1.6 and 2.4 kHz. The bottom figures visualize the four color encoded values of the four components of our two phi-bit representation over the entire range of tuning parameter (bottom left) and the focused region (bottom right) where the C-NOT operation is illustrated (see text for details).

Figure 1 illustrates the measured relative phases for phi-bits 13 and 14. Beside the spurious and unimportant 360° discontinuities, the two phases undergo a number of slow monotonous variations in addition to a number of resonant jumps of various magnitudes including 90 and 180°. The relative phases of phi-bits undergo multiple resonant and non-resonant scattering processes as a consequence of the non-linearities of the system. Some phi-bits exhibit only monotonous changes in phase, some of which extend over broad ranges of possible angles (not shown). We have verified the repeatability of the phase measurement and found some variations over several months. Although there is no limit on the stability of acoustic waves in the ultrasonic frequency domain nor on the accuracy of the measurement of phases of acoustic waves, the observed variations are due probably to aging of some components of the experimental set-up (e.g., organic ultrasonic coupling agent between rod and transducer and/or rubber-based rod-transducer attachments). In the current state of the apparatus, phase measurements appear to be conservatively accurate to 20 degrees.

**Figure 2:**
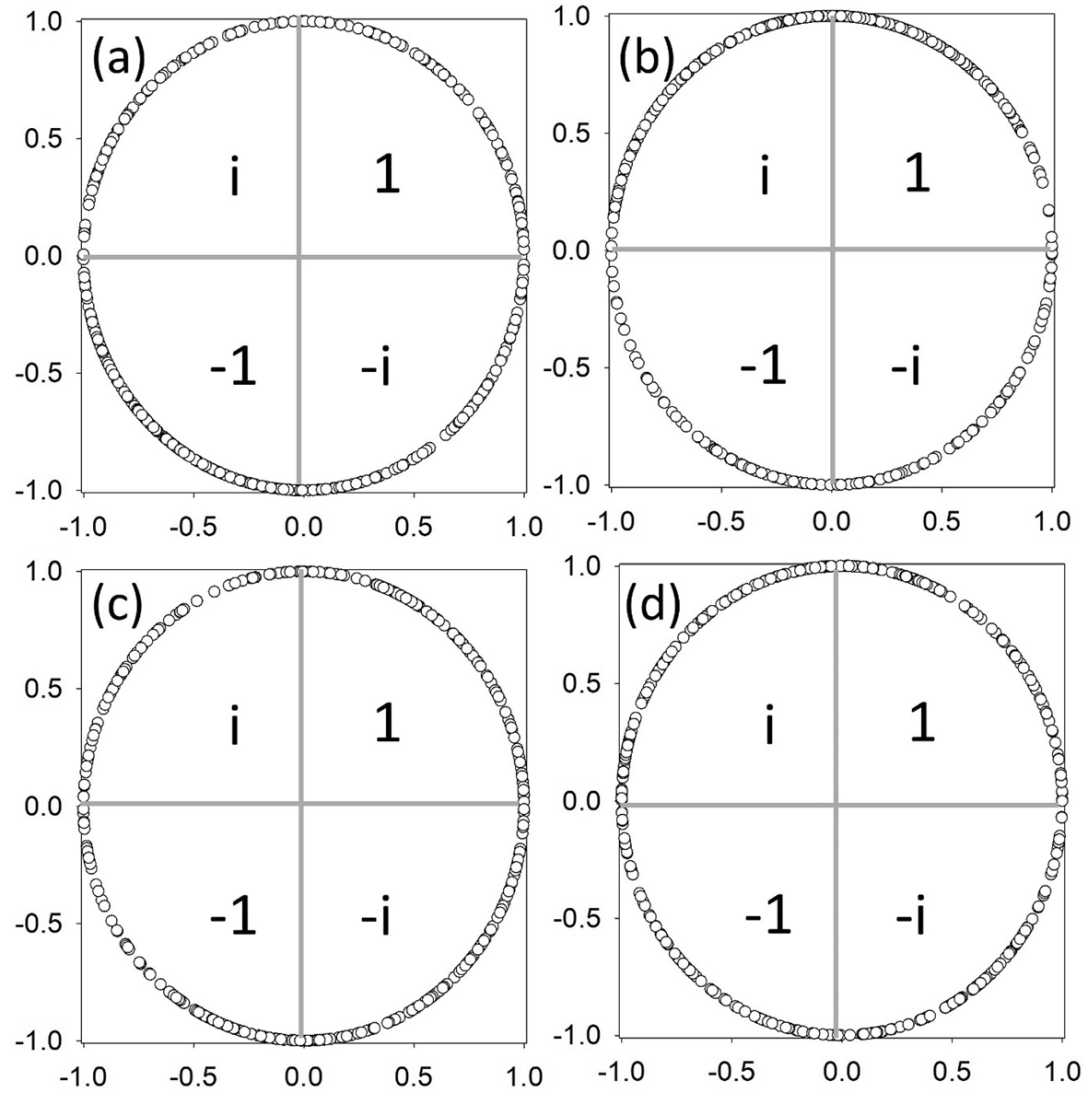
Two phi-bit states span the complete complex circle. Complex components, (**a**) $C_{11}^{ij}$, (**b**) $C_{12}^{ij}$, (**c**) $C_{21}^{ij}$, and (**d**) $C_{22}^{ij}$ for *i* = 13 and *j* = 14 represented on the complex unit circle for all 400 recorded values of the tuning parameter $\Delta \nu$. The circle is reduced to four quadrants corresponding to the values 1, i, − 1, and − i.

In Fig. 2, we show the components of $\vec{W}^{i = 13j = 14}$ for all recorded values of the tuning parameter in the case of the representation $\left\{ m = 2,n = 1,p = 3,q = 4  \right\}$ for the pair of phi-bits 13 and 14. Each complex coefficient covers well the unit circle. A few gaps are noticeable though. Because of the precision in the measurement of phase with our current apparatus and to simplify the search for a C-NOT unitary operation in the Hilbert space of pairs of phi-bits, we reduce the complex circle for each pair of phi-bits to four quarters and assign the values of 1, *i*, − 1, and − *i* to component data with 90° slices in the ranges [0°, 90°] for 1, [90°, 180°] for i, [180°, 270°] for − 1, and [270°, 0°] for −  I (the angles are measured in a counterclockwise direction from the x-axis). For each pair of phi-bits, the four vector $\vec{W}^{ij}$ can take 4^4^ = 256 possible values.

Unitary transformations such as the C-NOT gate can be represented by unitary matrices. In the case of two phi-bits, there are 6 such matrices of which the most common form is $$U_{CNOT} = \left( {\begin{array}{*{20}c} 1 & 0 & 0 & 0 \\ 0 & 1 & 0 & 0 \\ 0 & 0 & 0 & 1 \\ 0 & 0 & 1 & 0 \\ \end{array} } \right)$$. Other matrices are obtained by permutations of the diagonal and off-diagonal non-zero entries. We search for a transformation that when applied on one input, i.e., one of the 256 two phi-bit four vector states in their 4-dimensional Hilbert, produces systematically, reproducibly and predictably an output, i.e., its C-NOT transformed counterpart. That is, for each of the $P\left( {P - 1} \right) = 240$ pairs *ij*, we examine all possible 24 combinations of representations with coefficients $\left\{ {m,n,p,q} \right\}$ and all 6 possible C-NOT matrices for all values of the frequency tuning parameter. Of the 24 $\times 6 =$ 144 combinations of representations, C-NOT gates and for all pairs of phi-bits, one combination yields all 256 transformations for a given frequency change. That is the representation $\left\{ {m = 2,n = 1,p = 3,q = 4\} } \right\}$ and the common C-NOT matrix shown above. Furthermore, and most importantly, there is at least one one-frequency transformation that can be applied to every four-vector state to produce its C-NOT transformed counterpart, for instance by reducing $f_{1} - \Delta \nu$ by 100 Hz, i.e., increasing $\Delta \nu$ by 100 Hz.

For instance, the four vector $$\vec{W}^{i = 13j = 14} = \left( {\begin{array}{*{20}c} 1 \\ 1 \\ 1 \\ i \\ \end{array} } \right)$$ which is realized at $\Delta \nu = 1.89kHz$ transforms for the same pair of phi-bits to the vector $$\left( {\begin{array}{*{20}c} 1 \\ 1 \\ i \\ 1 \\ \end{array} } \right)$$ by simply changing the parameter $\Delta \nu$ to $1.99kHz$. We show this visually in the bottom right of Fig. 1, encoding the values using colors, where the red (*i*) value swaps from the fourth row to the third. Similarly (not shown visually), $$\vec{W}^{i = 5j = 6} \left( {\Delta \nu = 0.78kHz} \right) = \left( {\begin{array}{*{20}c} 1 \\ i \\ 1 \\ { - 1} \\ \end{array} } \right)$$ transforms to $$\vec{W}^{i = 5j = 6} \left( {\Delta \nu = 0.88kHz} \right) = \left( {\begin{array}{*{20}c} 1 \\ i \\ { - 1} \\ 1 \\ \end{array} } \right)$$. The complete list of four vectors and their corresponding phi-bit pairs and frequency tuning parameters are provided in the Supplementary Information.

Implementation of the C-NOT gate within the context of a chosen representation, would require a computer program, known in the quantum computing language as an oracle, which for a given four-vector input, would set up the physical system (i.e., set up the driving frequencies, frequency tuning parameter and corresponding pair of logical phi-bits, *ij*, which determine the set of measurable input phases: $\varphi_{12}^{i}$, $\varphi_{13}^{i}$, $\varphi_{12}^{j}$, $\varphi_{13}^{j}$). The C-NOT gate operation is simply conducted by reducing the driving frequency of the first waveguide by 100 Hz, this independently of the input. The oracle then can read the C-NOT transformed four-vector output from the phases associated with the new state of the same phi-bit pair. These phases are again directly measurable. This approach demonstrates that acoustic metamaterials can be used to operate on complex vectors in the Hilbert space of pairs of logical phi-bits to achieve a systematic and predictable C-NOT gate with unambiguously measurable input and output.

## Discussion

The approach and results presented here shows the feasibility and practicality of employing an externally driven nonlinear acoustic-metamaterial platform supporting logical phi-bit to realize a two-bit quantum-like C-NOT gate. The gate operation is achieved with a simple physical manipulation involving the detuning of the frequency of one of the external drivers. This approach is scalable as the apparatus can support exponentially complex Hilbert spaces of multiple phi-bits. With a conceivable increase in precision of the experimental set-up (e.g., by using non-aging materials and permanent attachments), we could span more states in the Hilbert space of multiple phi-bits. For instance, we can discretize the unit circle into 2^3^ = 8 parts instead of the four as demonstrated here. With the complete set of 16 logical phi-bits, we could represent 8^16^ or 2^48^ unique vectors in a 2^16^-dimensional Hilbert space. The nonlinear array of three acoustic waveguides with 2 drivers has therefore the data manipulation capacity of 48 fully classically entangled qubit-analogues. The remaining challenge is to identify simple protocols to navigate these Hilbert spaces very high dimensionality and achieve predictable operations analogous to multiple qubit quantum gates.

## Methods

### Physical platform

Three aluminum rods (McMaster-Carr multipurpose 6061 aluminum rod with certification 1/2" diameter, 0.609 m length, and density $\rho = 2,660$ ${\text{kg}}/{\text{m}}^{3}$) are used in the experimental realization of the nonlinear acoustic waveguide-transducer-amplifier-generator platform. Epoxy is used to fill the lateral gap between the rods (50,176 KwikWeld Syringe). The acoustic field at the rod ends is driven and detected by two sets of transducers (V133-RM—Olympus IMS). Through PD200 amplifiers (PD200 is a high bandwidth, low-noise linear amplifier), the two driving transducers are coupled to waveform generators (B&K Precision 4055B). To detect signals at the rod end, the three recording transducers are connected to a Tektronix oscilloscope (MDO3024). The oscilloscope records the input (driving) and output (response) signals. The waveform generators are connected to a computer for experiment control, and the oscilloscopes are likewise connected to a digital computer for data processing.

### C-NOT operation

The state of a phi-bit associated with a nonlinear acoustic mode, “*i*”, in an externally driven three-waveguide nonlinear system can be defined by a $2 \times 1$ vector representation:

$$ \vec{U}_{\left( i \right)} = \left( {\begin{array}{*{20}c} {\hat{c}_{2} e^{{i\varphi_{12}^{i} }} } \\ {\hat{c}_{3} e^{{i\varphi_{13}^{i} }} } \\ \end{array} } \right)e^{{i\omega^{\left( i \right)} t}} $$

where the nonlinear angular frequency $\omega^{\left( i \right)}$ is a mixture of the driving frequencies. The magnitudes $\hat{C}_{2}$ and $\hat{C}_{3}$ of waveguides 2 and 3 are normalized to that of guide 1, and $\varphi_{12}^{i} = \varphi_{2}^{i} - \varphi_{1}^{i}$ and $\varphi_{13}^{i} = \varphi_{3}^{i} - \varphi_{1}^{i}$ are the two independent phases in waveguides 2 and 3 relative to that of guide 1. Amplitude and phases are measured unambiguously at the ends of the waveguides. This single phi-bit state lives in a two-dimensional Hilbert space, $h_{\left( i \right)}$.

The state of a non-interacting *P* phi-bit system is the tensor products of single phi-bit states, namely: $\vec{W} = \vec{U}_{\left( 1 \right)} \otimes \ldots \otimes \vec{U}_{\left( P \right)}$. The tensor product of the basis vectors of single phi-bit forms a complete basis for the states of the noninteracting multi phi-bit system. This basis defines a $2^{P}$ dimensional Hilbert space, *H*. *H* is the tensor product of the *P* Hilbert spaces of the individual non-interacting phi-bits, $H = h_{\left( 1 \right)} \otimes \ldots \otimes h_{\left( P \right)}$. In the case of the nonlinear coupled waveguide system discussed here, phi-bits interact, and the Hilbert space spanned by the states of the interacting system is the same as for a noninteracting system. However, a state of the interacting system may now be a separable or non-separable linear combination (with complex coefficients) of the basis vectors of *H*. We can then define different representations of the *P* logical phi-bit system by applying unitary transformations to the basis of *H*. Here, we focus on representations leading to a multipartite tensor product structure that is conditioned by the measurability of the phases of each logical phi-bit. We may then choose a representation for its practicality in implementing quantum-like gates.

Since the C-NOT gate is a two-bit gate, we investigate representations involving two phi-bits among the 16 available^23^. We consider representations such that the complex coefficients of states of a system constituted of any two different phi-bits, “*i*” and “*j*” take the form: $$\vec{W}^{ij} = \left( {\begin{array}{*{20}c} {C_{11}^{ij} } \\ {C_{12}^{ij} } \\ {C_{21}^{ij} } \\ {C_{22}^{ij} } \\ \end{array} } \right) $$ where $C_{11}^{ij} = exp\left[ {mi\left( {\varphi_{12}^{i} + \varphi_{12}^{j} } \right)} \right]$, $C_{12}^{ij} = exp\left[ {ni\left( {\varphi_{13}^{i} + \varphi_{12}^{j} } \right)} \right]$, $C_{21}^{ij} = exp\left[ {pi\left( {\varphi_{12}^{i} + \varphi_{13}^{j} } \right)} \right]$, and $C_{22}^{ij} = exp\left[ {qi\left( {\varphi_{13}^{i} + \varphi_{13}^{j} } \right)} \right]$ and the set {*n*, *m*, *p*, *q*} are permutations of the integers, 1, 2, 3 and 4. Note that this representation differentiates the pairs *ij* and *ji*. There are 24 such representations for each pair of phi-bits. All live in the four-dimensional Hilbert spaces, $H = h_{\left( i \right)} \otimes h_{\left( j \right)}$.

In detail, the search for the C-NOT gate in the data collected is conducted in three steps. First, all phi-bit pairs are interrogated to determine which sets of tuning parameters correspond to C-NOT operations and these are stored in files labelled by the phi-bit pairs. These are subsequently parsed and reorganized by the initial 4-vector components labelled numerically from 0 to 255. Finally, the database of initial 4-vector components is analyzed to find a common set of tuning parameters, i.e., a single $\Delta \nu$, for which all C-NOT gate operations can be realized.

The complex coefficients of these representations, through the relative phases, are dependent on the driving frequencies $$f_{1}$$ and $$ f_{2}$$ and are therefore tunable. Here, we have taken $$f_{1} = 62 kHz$$ and $$f_{2} = 66 kHz$$ and chosen to modify $$f_{1} $$ in the form $$f_{1} - \Delta \nu$$ while keeping $$f_{2}$$ constant. We vary the frequency tuning parameter $$\Delta \nu$$ by increments of $$10Hz$$ up to $$4kHz$$.

## Supplementary Information

Supplementary Information.

## Data Availability

The data that support our findings of the present study are available from the corresponding author upon reasonable request.
