# Supplementary material for: Demonstration of a two-bit controlled-NOT quantum-like gate using classical acoustic qubit-analogues
Source: Sci Rep. 2022 Aug 18;12:14066. doi: 10.1038/s41598-022-18314-5 (PMC9388580; doi:10.1038/s41598-022-18314-5)
Supplement: Supplementary file 1 — Supplementary Information. [file 41598_2022_18314_MOESM1_ESM.pdf]

**Table 1: Phi-bit pairs and frequency tuning parameters**

Complete list of four vector components W_k^ij, their corresponding phi-bit pairs and frequency tuning parameters.
Tuning parameters are in kHz, waveguide 1 is driven at 62 kHz - Δν. Changing Δν by 0.1 kHz accomplishes the C-NOT gate operation.

| $W_1^{ij}$ | $W_2^{ij}$ | $W_3^{ij}$ | $W_4^{ij}$ | Phi-bit pair: *i* , *j* | Tuning parameter: $\Delta\nu$ |
|---|---|---|---|---|---|
| 1 | 1 | 1 | 1 | 1,7 | 3.88 |
| 1 | 1 | 1 | i | 1,13 | 1.11 |
| 1 | 1 | 1 | -1 | 12,3 | 0.38 |
| 1 | 1 | 1 | -i | 7,2 | 1.25 |
| 1 | 1 | i | 1 | 4,6 | 1.5 |
| 1 | 1 | i | i | 3,8 | 0 |
| 1 | 1 | i | -1 | 3,6 | 1.02 |
| 1 | 1 | i | -i | 1,3 | 1.95 |
| 1 | 1 | -1 | 1 | 3,12 | 0.38 |
| 1 | 1 | -1 | i | 1,4 | 1.02 |
| 1 | 1 | -1 | -1 | 3,10 | 3.55 |
| 1 | 1 | -1 | -i | 2,4 | 1.21 |
| 1 | 1 | -i | 1 | 1,4 | 2.56 |
| 1 | 1 | -i | i | 4,12 | 1.51 |
| 1 | 1 | -i | -1 | 4,2 | 1.21 |
| 1 | 1 | -i | -i | 3,13 | 3.7 |
| 1 | i | 1 | 1 | 2,9 | 1.9 |
| 1 | i | 1 | i | 6,15 | 2.57 |
| 1 | i | 1 | -1 | 4,15 | 3.08 |
| 1 | i | 1 | -i | 5,6 | 0.78 |
| 1 | i | i | 1 | 15,6 | 2.57 |
| 1 | i | i | i | 1,15 | 1.74 |

| | | | | | |
|---|---|---|---|---|---|
| 1 | i | i | -1 | 3,6 | 1.31 |
| 1 | i | i | -i | 1,13 | 0.78 |
| 1 | i | -1 | 1 | 15,4 | 3.08 |
| 1 | i | -1 | i | 6,3 | 1.31 |
| 1 | i | -1 | -1 | 4,16 | 0.32 |
| 1 | i | -1 | -i | 7,2 | 1.34 |
| 1 | i | -i | 1 | 1,14 | 3 |
| 1 | i | -i | i | 1,7 | 1.09 |
| 1 | i | -i | -1 | 2,7 | 1.34 |
| 1 | i | -i | -i | 2,7 | 0.66 |
| 1 | -1 | 1 | 1 | 4,16 | 1.49 |
| 1 | -1 | 1 | i | 5,6 | 1.36 |
| 1 | -1 | 1 | -1 | 2,4 | 1.42 |
| 1 | -1 | 1 | -i | 8,9 | 0.73 |
| 1 | -1 | i | 1 | 6,5 | 1.36 |
| 1 | -1 | i | i | 3,5 | 0.6 |
| 1 | -1 | i | -1 | 8,15 | 1.36 |
| 1 | -1 | i | -i | 3,11 | 0.82 |
| 1 | -1 | -1 | 1 | 4,2 | 1.42 |
| 1 | -1 | -1 | i | 15,8 | 1.36 |
| 1 | -1 | -1 | -1 | 9,14 | 3.29 |
| 1 | -1 | -1 | -i | 2,14 | 1.28 |
| 1 | -1 | -i | 1 | 9,8 | 0.73 |
| 1 | -1 | -i | i | 11,3 | 0.82 |
| 1 | -1 | -i | -1 | 14,2 | 1.28 |
| 1 | -1 | -i | -i | 2,15 | 2.47 |
| 1 | -i | 1 | 1 | 1,14 | 2.15 |
| 1 | -i | 1 | i | 1,6 | 1.84 |
| 1 | -i | 1 | -1 | 1,2 | 3.58 |
| 1 | -i | 1 | -i | 12,7 | 1.46 |
| 1 | -i | i | 1 | 13,3 | 1.38 |
| 1 | -i | i | i | 2,3 | 0.63 |
| 1 | -i | i | -1 | 3,7 | 1.44 |

| | | | | | |
|---|---|---|---|---|---|
| 1 | -i | i | -i | 2,8 | 1.74 |
| 1 | -i | -1 | 1 | 2,1 | 3.58 |
| 1 | -i | -1 | i | 7,3 | 1.44 |
| 1 | -i | -1 | -1 | 2,13 | 0.48 |
| 1 | -i | -1 | -i | 3,7 | 1.49 |
| 1 | -i | -i | 1 | 7,12 | 1.46 |
| 1 | -i | -i | i | 8,2 | 1.74 |
| 1 | -i | -i | -1 | 7,3 | 1.49 |
| 1 | -i | -i | -i | 4,9 | 0.51 |
| i | 1 | 1 | 1 | 2,8 | 2.53 |
| i | 1 | 1 | i | 10,7 | 2.7 |
| i | 1 | 1 | -1 | 4,1 | 1.22 |
| i | 1 | 1 | -i | 10,1 | 2.28 |
| i | 1 | i | 1 | 7,10 | 2.7 |
| i | 1 | i | i | 3,7 | 1.94 |
| i | 1 | i | -1 | 4,1 | 1.23 |
| i | 1 | i | -i | 1,16 | 1.61 |
| i | 1 | -1 | 1 | 1,4 | 1.22 |
| i | 1 | -1 | i | 1,4 | 1.23 |
| i | 1 | -1 | -1 | 7,5 | 3.18 |
| i | 1 | -1 | -i | 3,2 | 1.42 |
| i | 1 | -i | 1 | 1,2 | 3.81 |
| i | 1 | -i | i | 1,4 | 1.27 |
| i | 1 | -i | -1 | 2,3 | 1.42 |
| i | 1 | -i | -i | 3,4 | 1.98 |
| i | i | 1 | 1 | 5,10 | 2.9 |
| i | i | 1 | i | 2,13 | 1.4 |
| i | i | 1 | -1 | 3,15 | 0.25 |
| i | i | 1 | -i | 2,5 | 1.34 |
| i | i | i | 1 | 13,2 | 1.4 |
| i | i | i | i | 4,9 | 1.88 |
| i | i | i | -1 | 3,1 | 1.03 |
| i | i | i | -i | 2,7 | 1.49 |

| | | | | | |
|---|---|---|---|---|---|
| i | i | -1 | 1 | 15,3 | 0.25 |
| i | i | -1 | i | 1,3 | 1.03 |
| i | i | -1 | -1 | 5,2 | 1.64 |
| i | i | -1 | -i | 3,9 | 0.95 |
| i | i | -i | 1 | 1,13 | 2.51 |
| i | i | -i | i | 7,2 | 1.49 |
| i | i | -i | -1 | 9,3 | 0.95 |
| i | i | -i | -i | 2,12 | 3.82 |
| i | -1 | 1 | 1 | 1,8 | 2.59 |
| i | -1 | 1 | i | 2,12 | 1.09 |
| i | -1 | 1 | -1 | 11,4 | 1.23 |
| i | -1 | 1 | -i | 6,2 | 1.16 |
| i | -1 | i | 1 | 12,2 | 1.09 |
| i | -1 | i | i | 4,8 | 0.89 |
| i | -1 | i | -1 | 5,12 | 1.1 |
| i | -1 | i | -i | 12,3 | 1.57 |
| i | -1 | -1 | 1 | 4,11 | 1.23 |
| i | -1 | -1 | i | 12,5 | 1.1 |
| i | -1 | -1 | -1 | 3,5 | 3.51 |
| i | -1 | -1 | -i | 2,6 | 1.14 |
| i | -1 | -i | 1 | 2,6 | 1.16 |
| i | -1 | -i | i | 1,2 | 1.19 |
| i | -1 | -i | -1 | 6,2 | 1.14 |
| i | -1 | -i | -i | 2,15 | 2.58 |
| i | -i | 1 | 1 | 1,2 | 2.38 |
| i | -i | 1 | i | 11,4 | 1.41 |
| i | -i | 1 | -1 | 11,3 | 1.02 |
| i | -i | 1 | -i | 2,3 | 1.18 |
| i | -i | i | 1 | 4,11 | 1.41 |
| i | -i | i | i | 2,3 | 0.66 |
| i | -i | i | -1 | 6,1 | 1.93 |
| i | -i | i | -i | 4,2 | 3.51 |
| i | -i | -1 | 1 | 3,11 | 1.02 |

| | | | | | |
|---|---|---|---|---|---|
| i | -i | -1 | i | 1,6 | 1.93 |
| i | -i | -1 | -1 | 2,5 | 0.63 |
| i | -i | -1 | -i | 3,2 | 1.12 |
| i | -i | -i | 1 | 3,2 | 1.18 |
| i | -i | -i | i | 2,4 | 3.51 |
| i | -i | -i | -1 | 2,3 | 1.12 |
| i | -i | -i | -i | 2,16 | 0.7 |
| -1 | 1 | 1 | 1 | 3,4 | 2.19 |
| -1 | 1 | 1 | i | 3,8 | 1.25 |
| -1 | 1 | 1 | -1 | 10,5 | 0.85 |
| -1 | 1 | 1 | -i | 11,3 | 1.29 |
| -1 | 1 | i | 1 | 8,3 | 1.25 |
| -1 | 1 | i | i | 8,9 | 2.64 |
| -1 | 1 | i | -1 | 7,1 | 1.49 |
| -1 | 1 | i | -i | 1,5 | 2.04 |
| -1 | 1 | -1 | 1 | 5,10 | 0.85 |
| -1 | 1 | -1 | i | 1,7 | 1.49 |
| -1 | 1 | -1 | -1 | 8,13 | 0.44 |
| -1 | 1 | -1 | -i | 7,1 | 1.56 |
| -1 | 1 | -i | 1 | 3,11 | 1.29 |
| -1 | 1 | -i | i | 4,3 | 3.24 |
| -1 | 1 | -i | -1 | 1,7 | 1.56 |
| -1 | 1 | -i | -i | 3,5 | 2.42 |
| -1 | i | 1 | 1 | 8,11 | 1.87 |
| -1 | i | 1 | i | 10,14 | 1.52 |
| -1 | i | 1 | -1 | 5,11 | 1.26 |
| -1 | i | 1 | -i | 11,3 | 1.54 |
| -1 | i | i | 1 | 14,10 | 1.52 |
| -1 | i | i | i | 7,1 | 2.64 |
| -1 | i | i | -1 | 2,9 | 2.31 |
| -1 | i | i | -i | 1,5 | 2.17 |
| -1 | i | -1 | 1 | 5,11 | 1.17 |
| -1 | i | -1 | i | 9,2 | 2.31 |

| | | | | | |
|---|---|---|---|---|---|
| -1 | i | -1 | -1 | 6,1 | 0.42 |
| -1 | i | -1 | -i | 3,1 | 1.06 |
| -1 | i | -i | 1 | 3,11 | 1.54 |
| -1 | i | -i | i | 4,9 | 2.1 |
| -1 | i | -i | -1 | 1,3 | 1.06 |
| -1 | i | -i | -i | 4,14 | 2.41 |
| -1 | -1 | 1 | 1 | 2,15 | 3.82 |
| -1 | -1 | 1 | i | 1,2 | 0.83 |
| -1 | -1 | 1 | -1 | 5,8 | 1.13 |
| -1 | -1 | 1 | -i | 9,14 | 2.13 |
| -1 | -1 | i | 1 | 2,1 | 0.83 |
| -1 | -1 | i | i | 2,6 | 2.66 |
| -1 | -1 | i | -1 | 9,2 | 0.83 |
| -1 | -1 | i | -i | 4,2 | 2.95 |
| -1 | -1 | -1 | 1 | 8,5 | 1.13 |
| -1 | -1 | -1 | i | 1,8 | 0.95 |
| -1 | -1 | -1 | -1 | 7,8 | 1.31 |
| -1 | -1 | -1 | -i | 9,2 | 0.92 |
| -1 | -1 | -i | 1 | 1,13 | 2.01 |
| -1 | -1 | -i | i | 2,4 | 2.95 |
| -1 | -1 | -i | -1 | 2,9 | 0.92 |
| -1 | -1 | -i | -i | 12,14 | 3.18 |
| -1 | -i | 1 | 1 | 3,14 | 0.6 |
| -1 | -i | 1 | i | 2,12 | 1.32 |
| -1 | -i | 1 | -1 | 4,8 | 1.83 |
| -1 | -i | 1 | -i | 9,2 | 1.3 |
| -1 | -i | i | 1 | 12,2 | 1.32 |
| -1 | -i | i | i | 1,14 | 0.34 |
| -1 | -i | i | -1 | 2,6 | 1.37 |
| -1 | -i | i | -i | 3,11 | 0.27 |
| -1 | -i | -1 | 1 | 8,4 | 1.83 |
| -1 | -i | -1 | i | 6,2 | 1.37 |
| -1 | -i | -1 | -1 | 2,7 | 2.37 |

| | | | | | |
|---|---|---|---|---|---|
| -1 | -i | -1 | -i | 6,2 | 1.39 |
| -1 | -i | -i | 1 | 2,9 | 1.3 |
| -1 | -i | -i | i | 11,3 | 0.27 |
| -1 | -i | -i | -1 | 2,6 | 1.39 |
| -1 | -i | -i | -i | 2,4 | 2.63 |
| -i | 1 | 1 | 1 | 1,3 | 2.55 |
| -i | 1 | 1 | i | 4,8 | 2.08 |
| -i | 1 | 1 | -1 | 11,2 | 3.63 |
| -i | 1 | 1 | -i | 1,5 | 2.72 |
| -i | 1 | i | 1 | 8,4 | 2.08 |
| -i | 1 | i | i | 5,4 | 1.22 |
| -i | 1 | i | -1 | 6,8 | 0.34 |
| -i | 1 | i | -i | 1,2 | 1.97 |
| -i | 1 | -1 | 1 | 2,11 | 3.63 |
| -i | 1 | -1 | i | 8,6 | 0.34 |
| -i | 1 | -1 | -1 | 2,8 | 3.9 |
| -i | 1 | -1 | -i | 2,1 | 1.36 |
| -i | 1 | -i | 1 | 6,11 | 1.51 |
| -i | 1 | -i | i | 5,10 | 1.51 |
| -i | 1 | -i | -1 | 1,10 | 0.51 |
| -i | 1 | -i | -i | 5,4 | 1.71 |
| -i | i | 1 | 1 | 16,9 | 3.39 |
| -i | i | 1 | i | 4,13 | 1.32 |
| -i | i | 1 | -1 | 7,4 | 3.07 |
| -i | i | 1 | -i | 14,2 | 1.02 |
| -i | i | i | 1 | 13,4 | 1.32 |
| -i | i | i | i | 1,11 | 1.82 |
| -i | i | i | -1 | 2,8 | 1.08 |
| -i | i | i | -i | 3,8 | 1.74 |
| -i | i | -1 | 1 | 4,7 | 3.07 |
| -i | i | -1 | i | 1,10 | 0.76 |
| -i | i | -1 | -1 | 3,7 | 3.85 |
| -i | i | -1 | -i | 6,4 | 1.15 |

| | | | | | |
|---|---|---|---|---|---|
| -i | i | -i | 1 | 2,14 | 1.02 |
| -i | i | -i | i | 8,3 | 1.74 |
| -i | i | -i | -1 | 4,6 | 1.15 |
| -i | i | -i | -i | 3,4 | 0.65 |
| -i | -1 | 1 | 1 | 2,4 | 2.14 |
| -i | -1 | 1 | i | 3,13 | 1.07 |
| -i | -1 | 1 | -1 | 8,5 | 1.3 |
| -i | -1 | 1 | -i | 9,16 | 1.96 |
| -i | -1 | i | 1 | 13,3 | 1.07 |
| -i | -1 | i | i | 2,6 | 0.14 |
| -i | -1 | i | -1 | 14,6 | 1.17 |
| -i | -1 | i | -i | 1,2 | 0.9 |
| -i | -1 | -1 | 1 | 5,8 | 1.3 |
| -i | -1 | -1 | i | 6,14 | 1.17 |
| -i | -1 | -1 | -1 | 4,6 | 0.02 |
| -i | -1 | -1 | -i | 3,7 | 1.14 |
| -i | -1 | -i | 1 | 16,9 | 1.96 |
| -i | -1 | -i | i | 2,1 | 0.9 |
| -i | -1 | -i | -1 | 7,3 | 1.14 |
| -i | -1 | -i | -i | 2,5 | 2.32 |
| -i | -i | 1 | 1 | 3,14 | 0.66 |
| -i | -i | 1 | i | 4,6 | 1.23 |
| -i | -i | 1 | -1 | 7,5 | 0.86 |
| -i | -i | 1 | -i | 1,2 | 1.16 |
| -i | -i | i | 1 | 6,4 | 1.23 |
| -i | -i | i | i | 1,9 | 0.67 |
| -i | -i | i | -1 | 3,6 | 2 |
| -i | -i | i | -i | 3,1 | 1.62 |
| -i | -i | -1 | 1 | 5,7 | 0.86 |
| -i | -i | -1 | i | 6,3 | 2 |
| -i | -i | -1 | -1 | 2,16 | 2.72 |
| -i | -i | -1 | -i | 6,14 | 1.2 |
| -i | -i | -i | 1 | 2,1 | 1.16 |

| | | | | | |
|---|---|---|---|---|---|
| -i | -i | -i | i | 1,3 | 1.62 |
| -i | -i | -i | -1 | 14,6 | 1.2 |
| -i | -i | -i | -i | 4,6 | 3.7 |
